# Supplementary material for: Direct conversion of cardiac fibroblasts into endothelial-like cells using Sox17 and Erg
Source: Nat Commun. 2024 May 16;15:4170. doi: 10.1038/s41467-024-48354-6 (PMC11098819; doi:10.1038/s41467-024-48354-6)
Supplement: Supplementary file 1 — Supplementary Information [file 41467_2024_48354_MOESM1_ESM.pdf]

Supplementary Figure 1: Additional Factor Screen Images

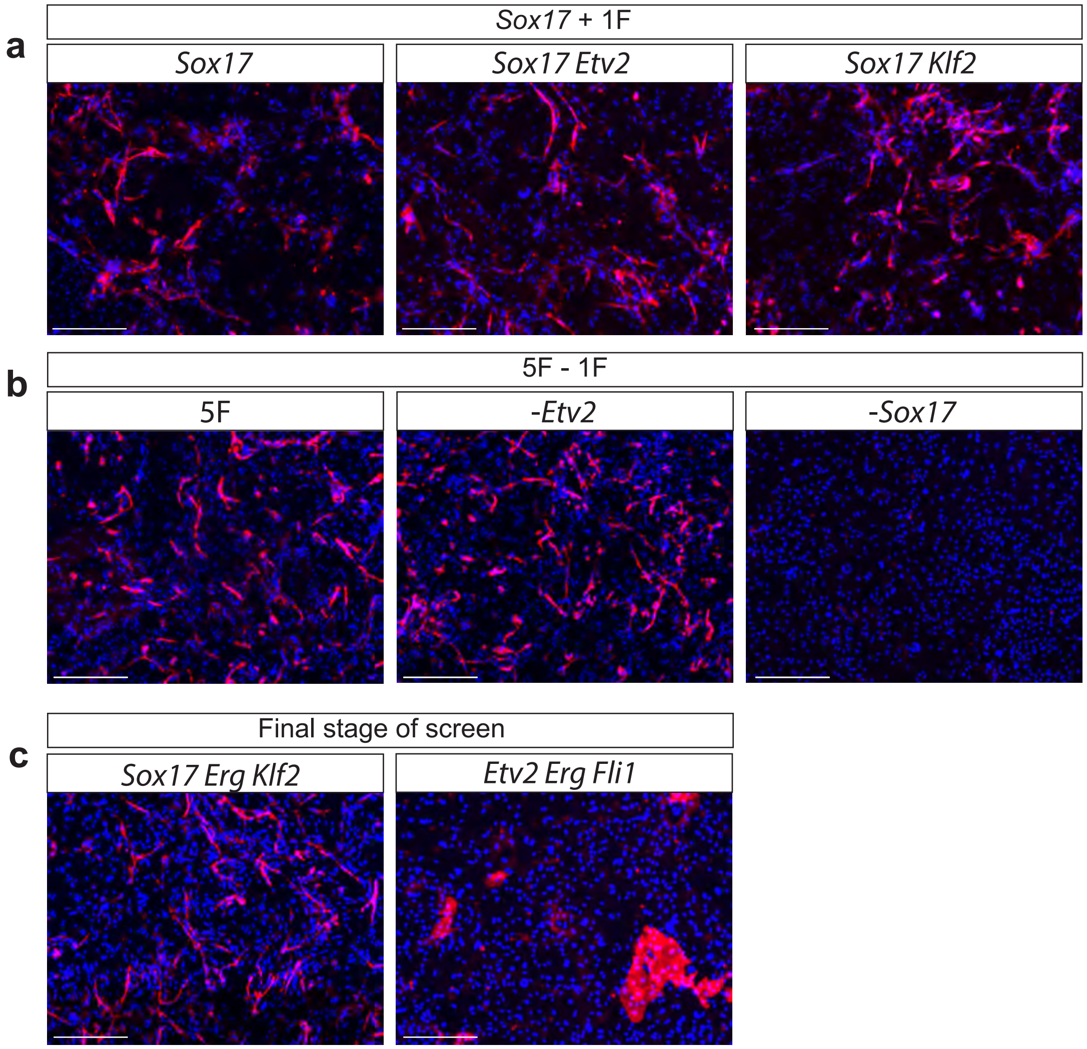

a, Additional images from *Sox17* + 1F stage of factor screen (scale bar 275 microns). **b**, Additional images from 5F-1 stage of factor screen (scale bar 275 microns). **c**, Additional images from final stage of screen. *Sox17 Erg Klf2* was also statistically significant in generating iECs when compared to *Sox17* alone. (scale bar 275 microns).

Supplementary Figure 2: Flow Cytometry Analysis of Depletion of PECAM1+ Cells during MACS isolation in Day 7 neonatal cardiac fibroblasts

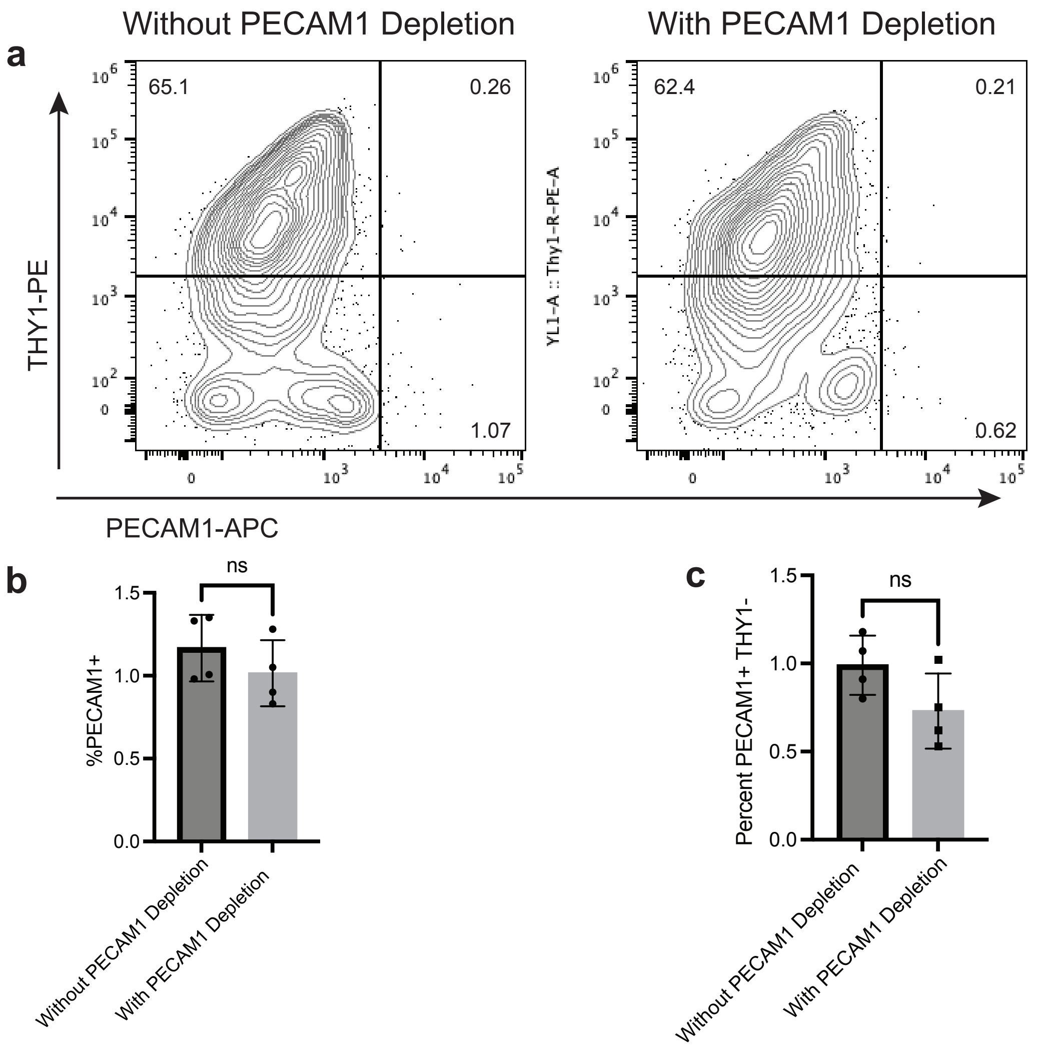

a, Flow cytometry plots of Day 7 samples stained with PECAM1 (x-axis) and THY1 (y-axis). **b**, Bar graph of percent PECAM1-positive cells. (two-sided student t-test, mean ± SD, n=4 samples)
c, Bar graph of percent PECAM1-positive THY1-negative cells. (two-sided student t-test, mean ± SD, n=4 samples) Source data are provided as a Source Data file.

Supplementary Figure 3: In vitro functional characterization images

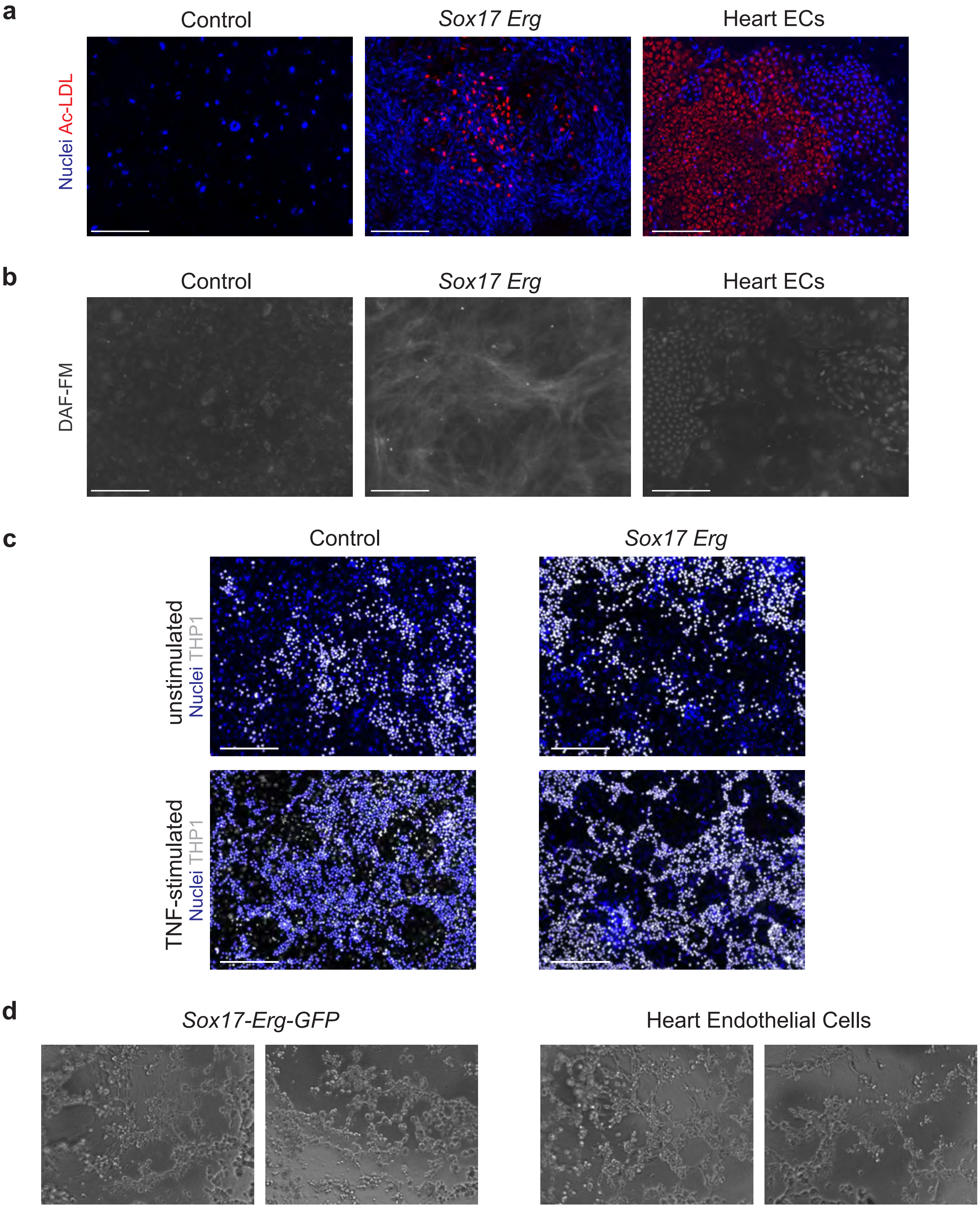

a, Representative Dil-Ac-LDL uptake images of four week old samples (scale bar 275 microns). **b**, Representative DAF-FM NO Production images (scale bar 275 microns). **c**, Representative images of unstimulated and TNFalpha stimulated iECs co-cultured with THP1 cells (scale bar 275 microns). **d**, Cord formation assay after 4 hours of incubation. **d**, Representative images of cord formation assay of SEG and native heart endothelial cells after 4 hours of culture on matrigel.

Supplementary Figure 4

a

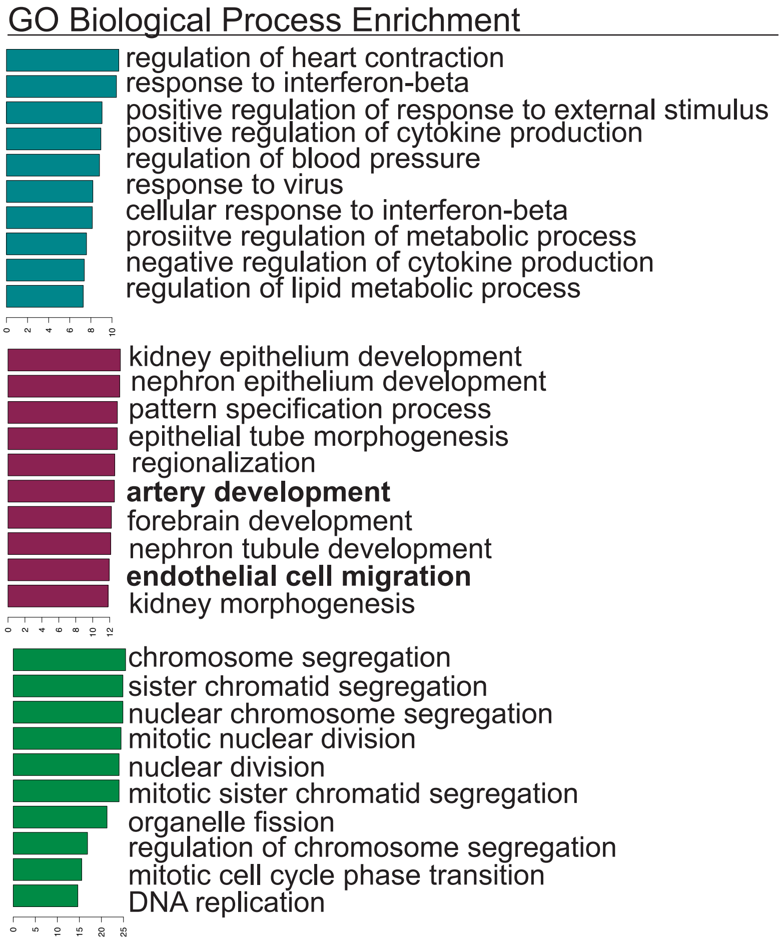

b

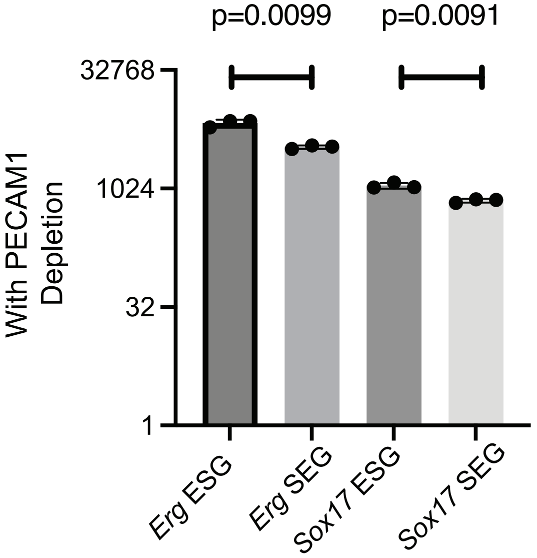

a, GO Biological Process Enrichment Analysis of Bulk RNAseq differentially expressed genes. **b**, qPCR of *Erg* and *Sox17* gene expression in *Erg-Sox17-GFP* (ESG) and *Sox17-Erg-GFP* (SEG) Day 7 construct samples. (n= 3 samples, two-sided student t-test, mean ± SD). Source data are provided as a Source Data file.

Supplementary Figure 5: In vivo functional characterization

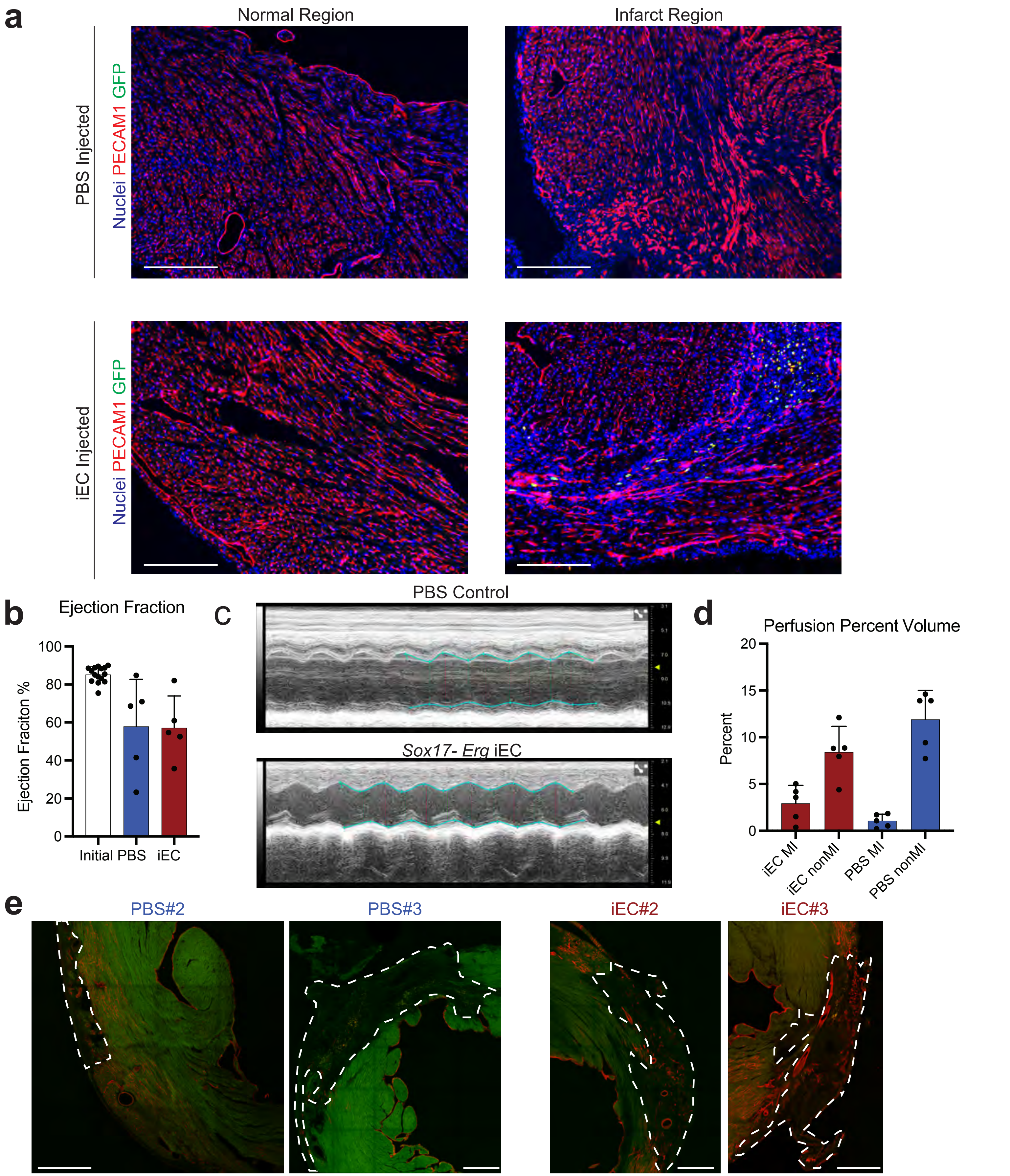

a, 10 micron thick sections of Day 7 infarcted hearts from PBS-control and iEC cell-injected samples (scale bar 275 microns). **b**, Ejection Fraction Quantification of four-week samples. (initial n=15 animals, PBS and iEC n =5 animals, mean ± SD) **c**, Example echocardiography images from 4 week timepoint mice **d**, Quantification of Perfusion Volume in scar and non-scar regions in four-week samples. **e**, Additional images of lectin perfused scar regions from different samples (scale bar 500 microns). Source data are provided as a Source Data file.

Supplementary Figure 6: scRNAseq Quality Control Metrics

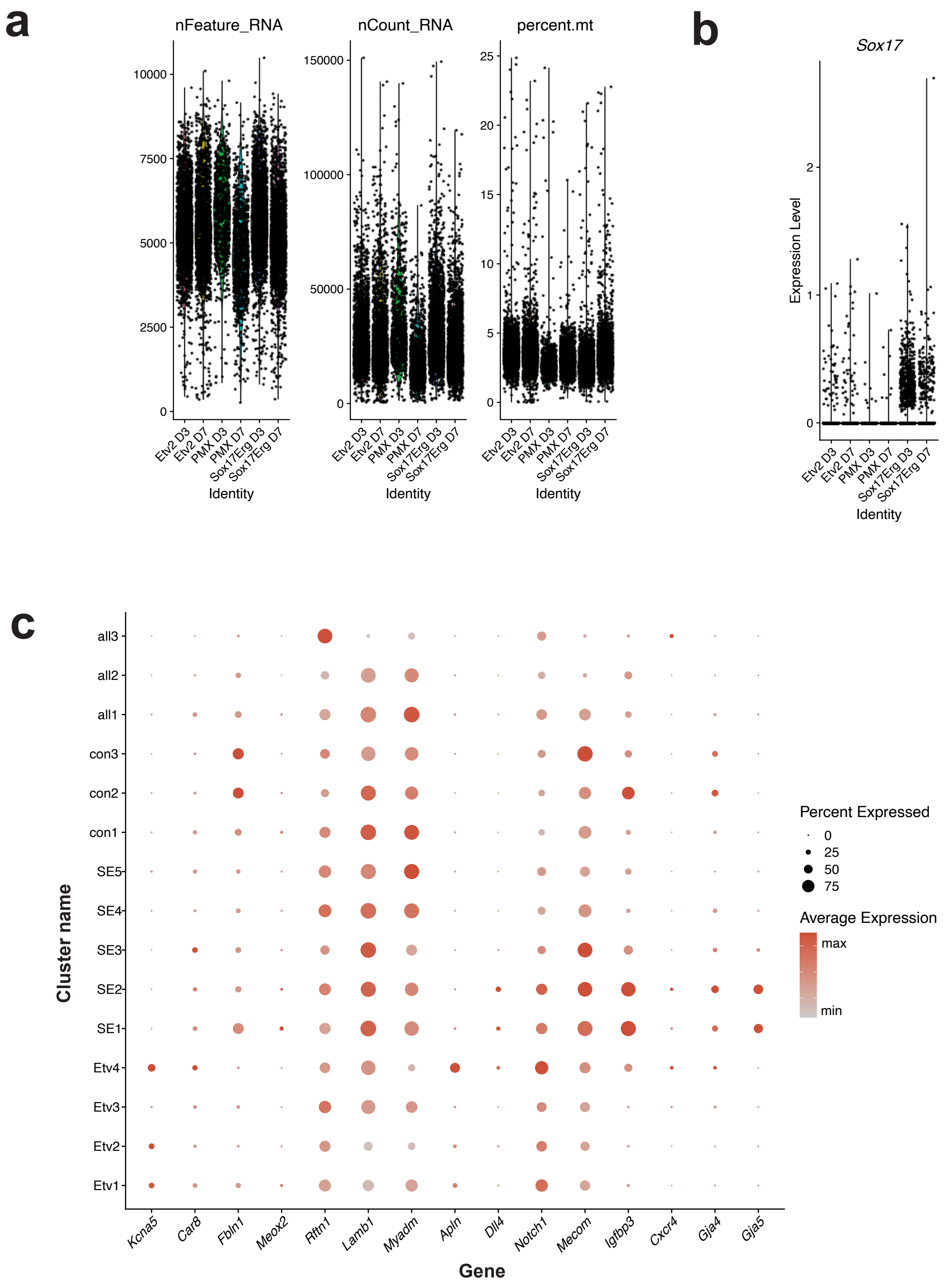

a, Quality control metrics of demultiplexed and combined Seurat object of scRNAseq data. **b**, Violin plots of gene expression of reprogramming genes annotated by sample. **c,** Dot plot of curated list of heart-specific endothelial and arterial genes.

Supplementary Figure 7: scRNAseq Cluster-specific Signaling Patterns

a

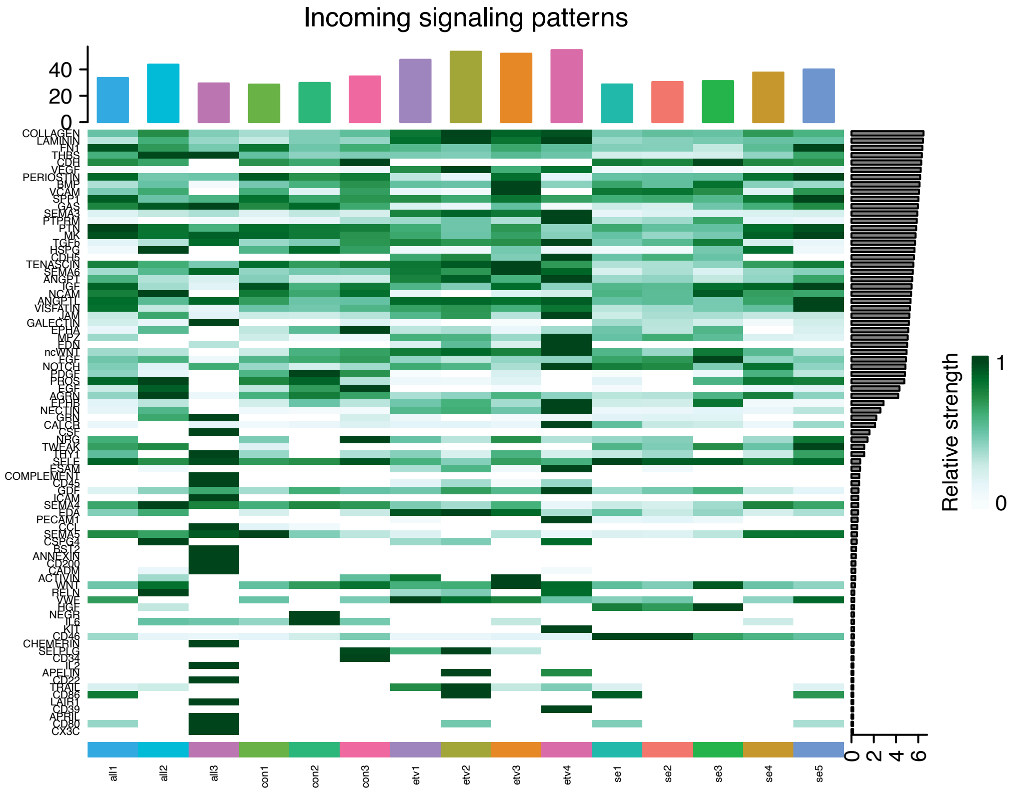

b

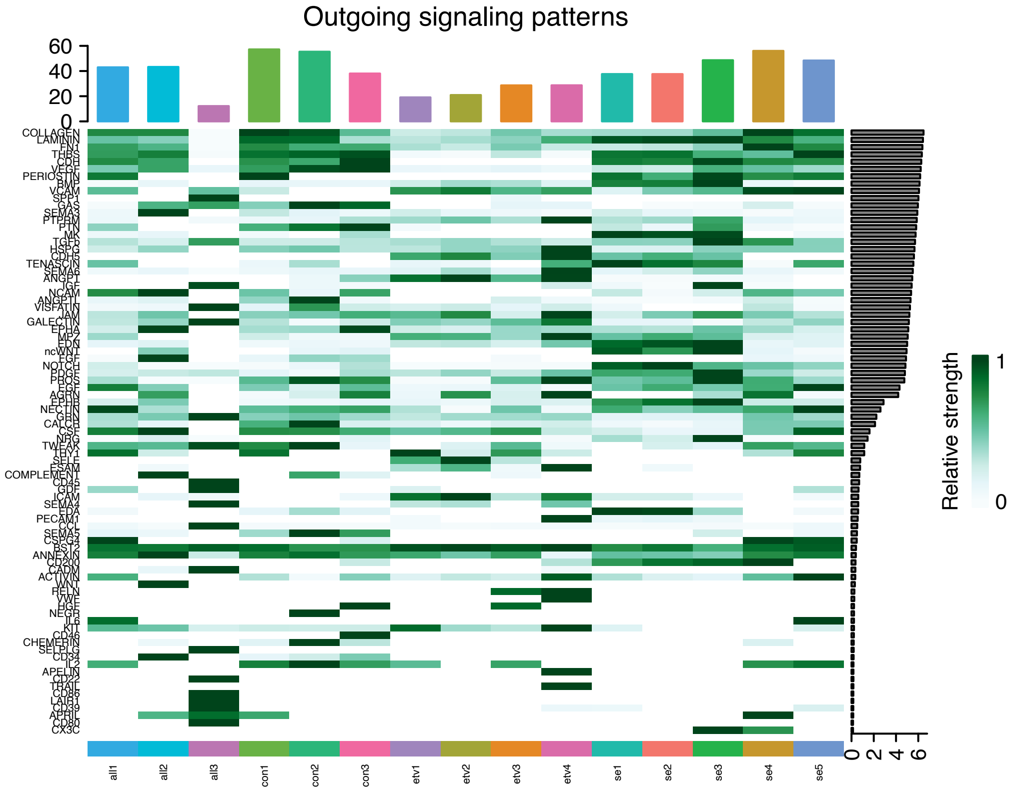

a, **b**, Predicted incoming and outgoing cell signaling pathways associated with specific clusters.

Supplementary Figure 8: Multi-Organ Fibroblast Reprogramming

a

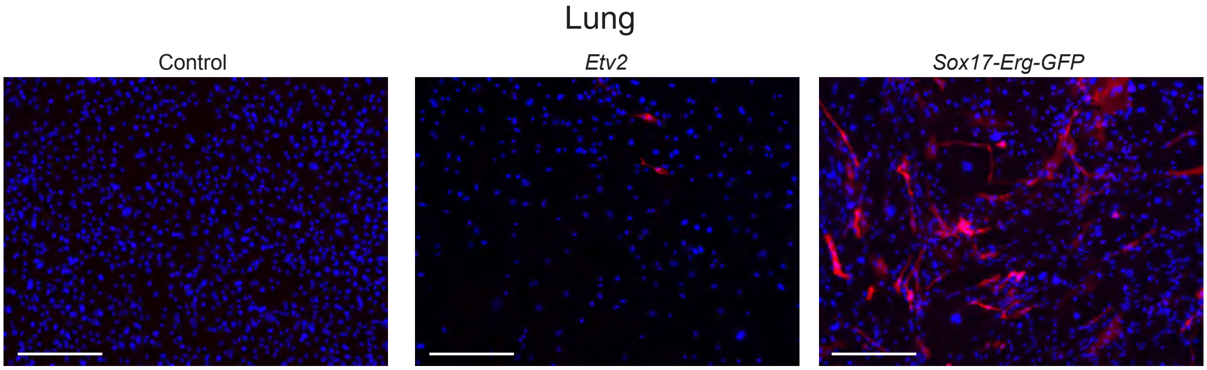

b

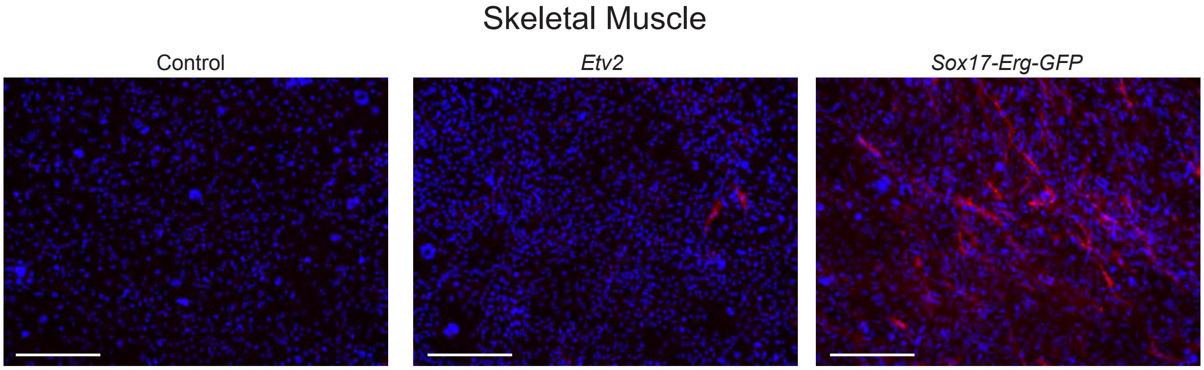

c

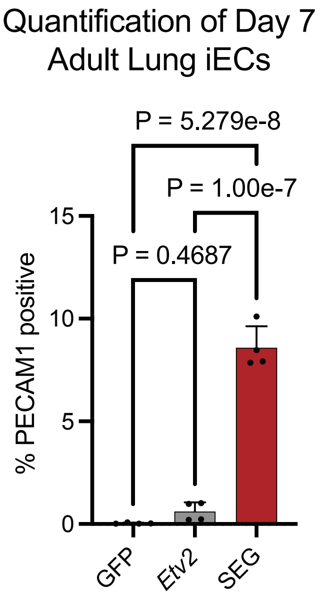

d

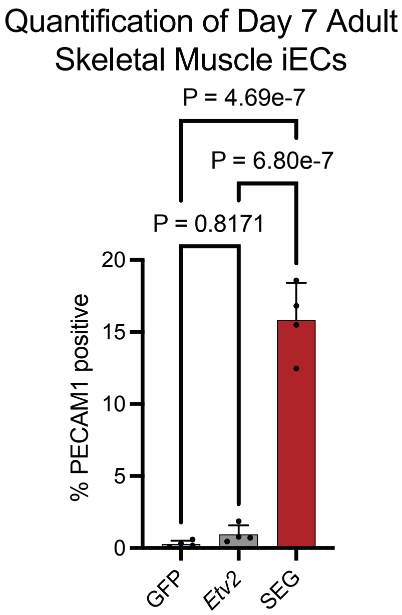

a, Images of Day 7 iEC reprogramming of isolated adult murine lung Thy1+ fibroblasts stained with Pecam (in red) and DAPI (in blue) (scale bar: 275 microns). **b**, Images of Day 7 iEC reprogramming of isolated adult murine skeletal muscle Thy1+ fibroblasts stained with Pecam (in red) and DAPI (in blue) (scale bar: 275 microns). **c, d,** Quantification of Day 7 Lung (c) and Skeletal Pecam-positive iECs (n=4 samples). (two-sided student t-test, mean ± SD). Source data are provided as a Source Data file.

Supplementary Figure 9: CUT&Tag Analysis Metrics

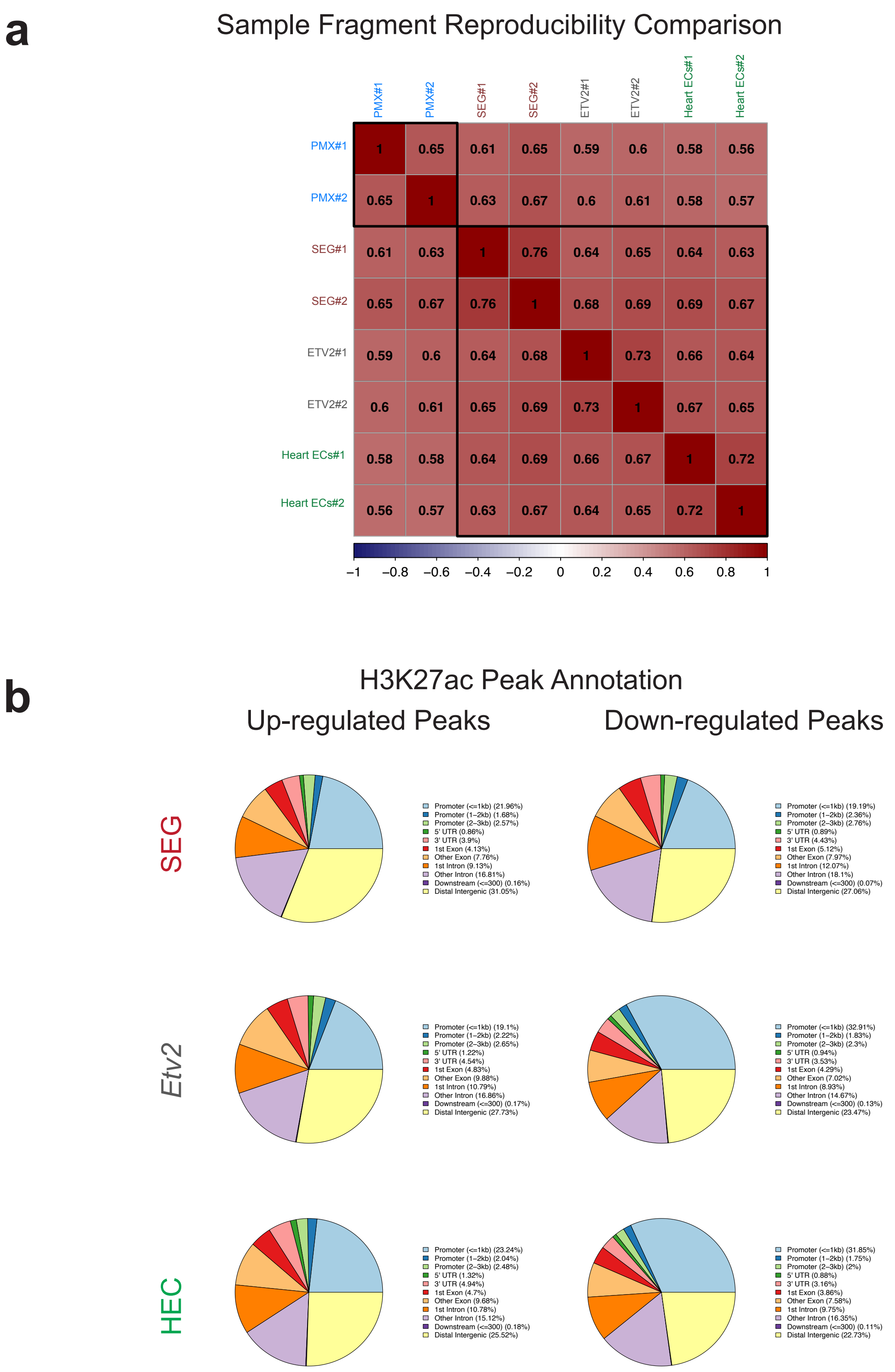

a, H3K27ac CUT&Tag fragment reproducibility comparisons between samples. **b**, H3K27ac Peak Gene Annotation metrics

Supplementary Figure 10: Peaks of Cluster-specific Marker Genes

a

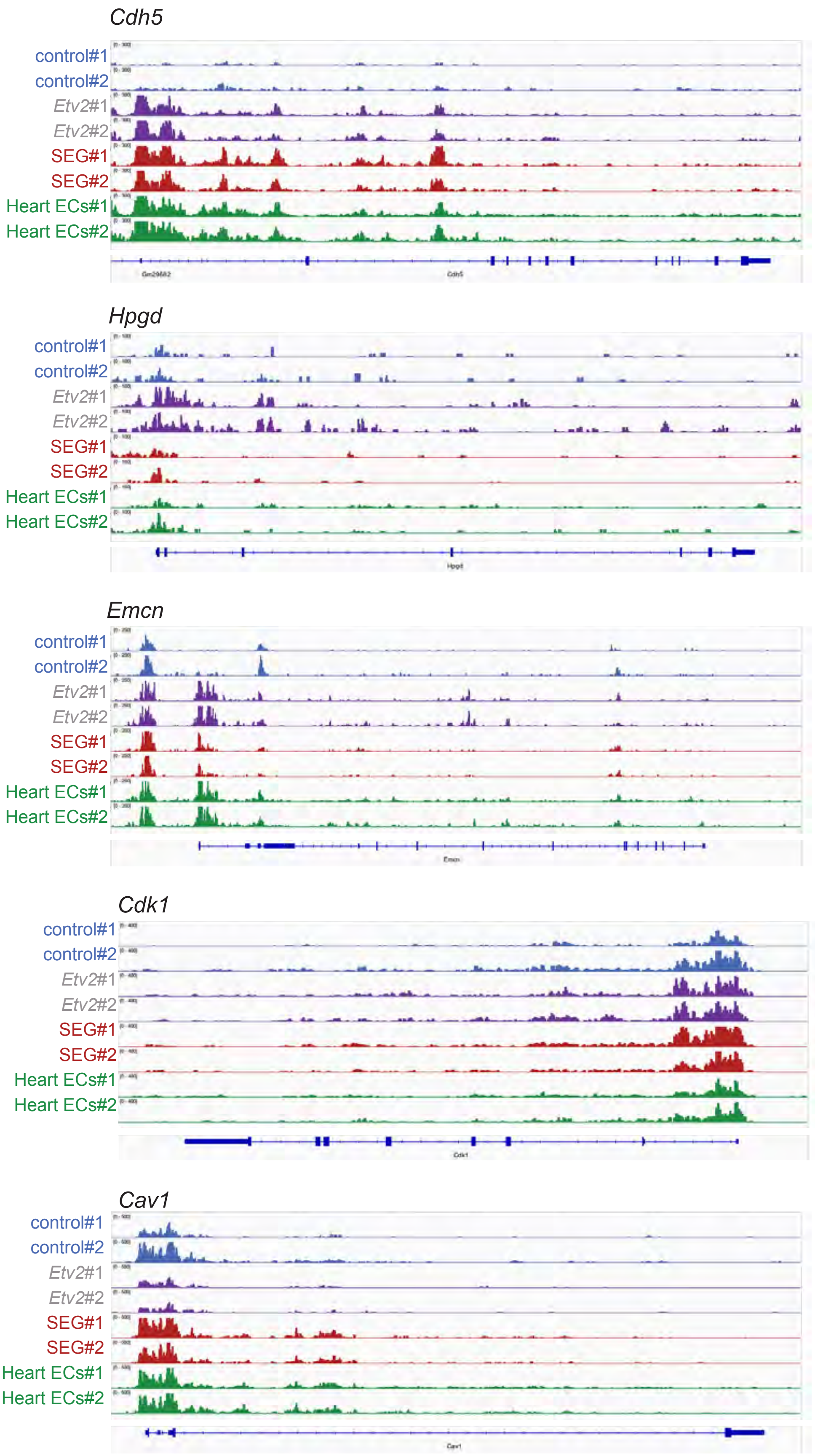

a, H3K27ac peaks in selected cluster-specific marker genes

Supplementary Figure 11: qPCR Comparison of Etv2 and SEG iECs to isolated adult murine heart endothelial cells

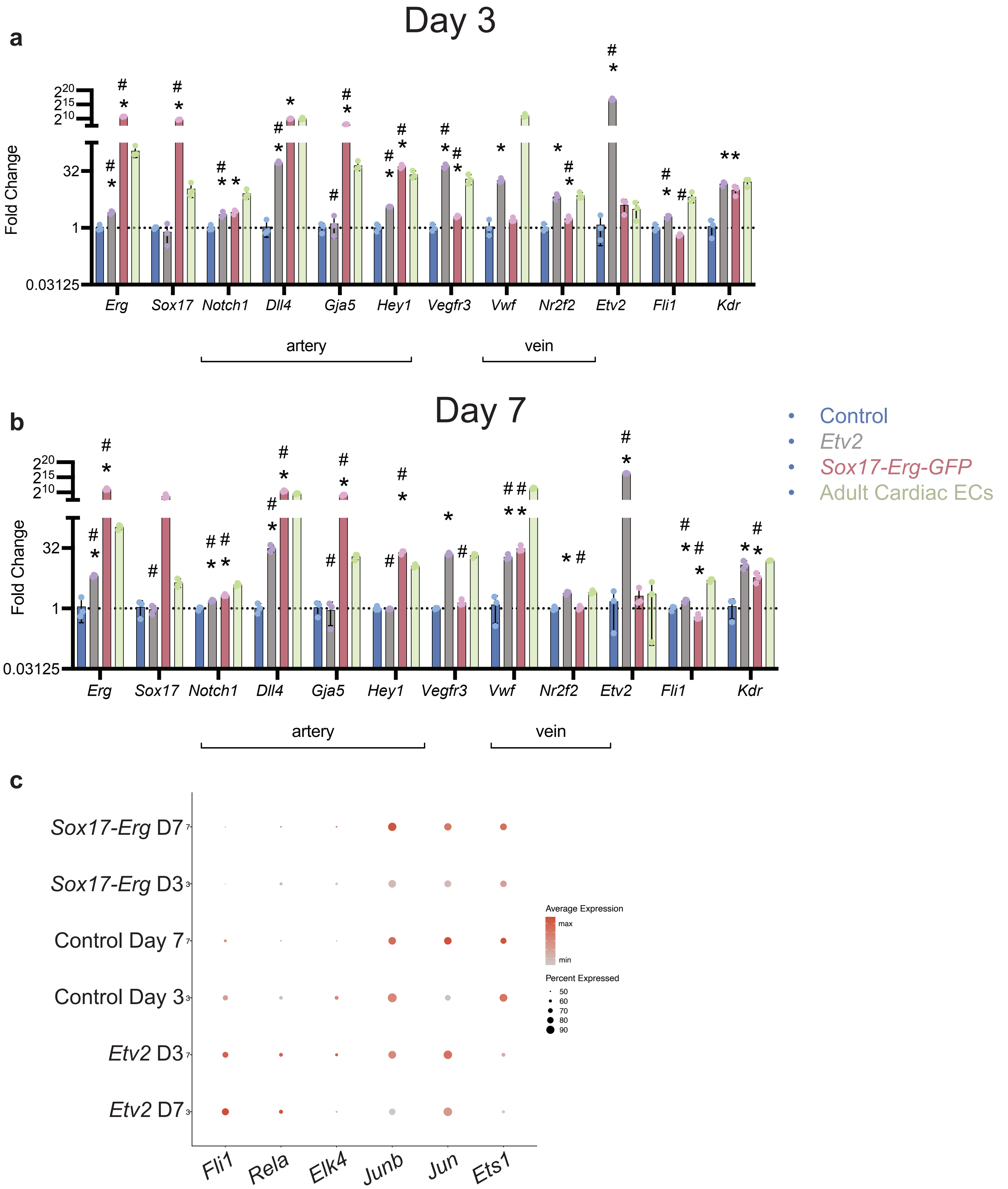

a, qPCR of Day 3 control, Etv2, SEG, and murine adult heart endothelial cells. (students t-test, mean ±SD) **b**, qPCR of Day 7 control, Etv2, SEG, and murine adult heart endothelial cells. (students t-test, mean ±SD) *p-value < 0.05 for comparison to control cells. #p-value <0.05 for comparison to heart endothelial cells. **c**, scRNAseq Dot plot of top hits from HOMER analysis of H3K27ac analysis. Source data are provided as a Source Data file.

Supplementary Figure 12: adult iEC scRNAseq

a

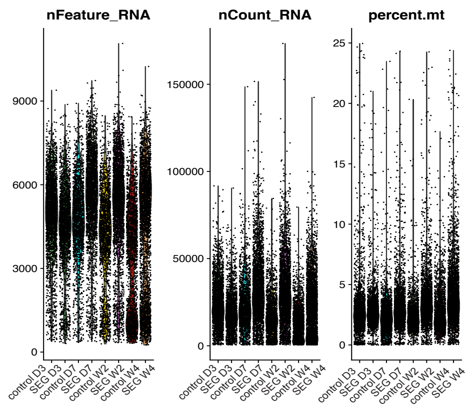

b

	control#1	control#2	control#3	control#4	control#5	control#6	SEG#1	SEG#2	SEG#3	SEG#4	SEG#5	SEG#6	generic#1	generic#2
control D3	**59.3**	**22.6**	**7.3**	1.1	0.5	0.3	0.0	3.1	0.0	0.1	0.2	1.0	2.8	1.6
control D7	2.4	10.9	0.6	**65.5**	10.9	2.2	0.0	0.7	0.1	0.1	0.9	2.5	2.2	1.0
control W2	0.2	1.0	1.0	17.2	**58.4**	15.6	0.0	0.6	0.2	0.1	0.7	3.1	0.8	1.2
control W4	0.1	0.5	0.6	7.5	49.9	**35.7**	0.2	0.5	0.4	0.0	0.5	2.4	0.9	0.6
SEG D3	3.9	5.9	0.8	0.2	0.2	0.1	**46.2**	**9.6**	10.4	**10.2**	5.0	5.6	1.3	0.6
SEG D7	0.8	4.8	0.1	2.3	0.3	1.0	18.0	3.5	28.4	8.0	17.7	11.5	2.6	0.8
SEG W2	0.2	0.9	0.1	1.7	1.4	5.3	8.9	3.4	**36.1**	1.7	**21.4**	**16.1**	1.5	1.4
SEG W4	0.2	0.1	0.2	1.2	2.8	21.5	8.3	5.6	26.4	0.6	15.6	12.5	2.4	2.6

e

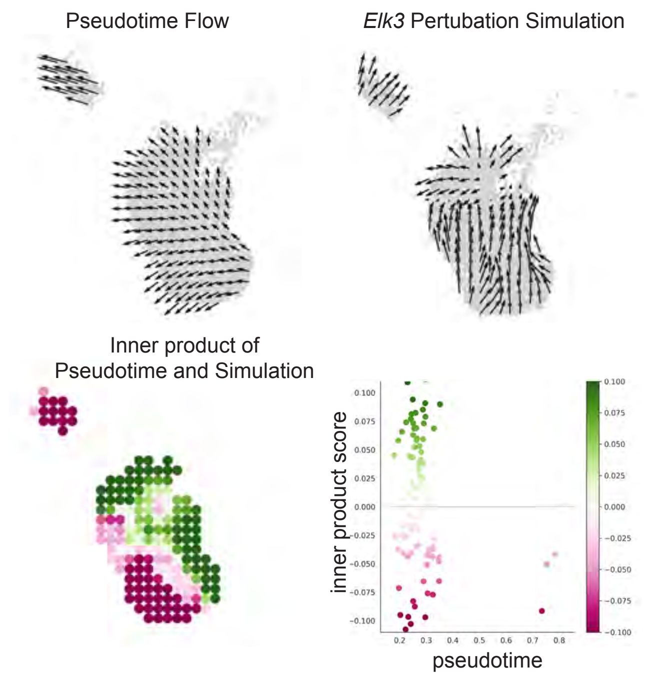

c **d**

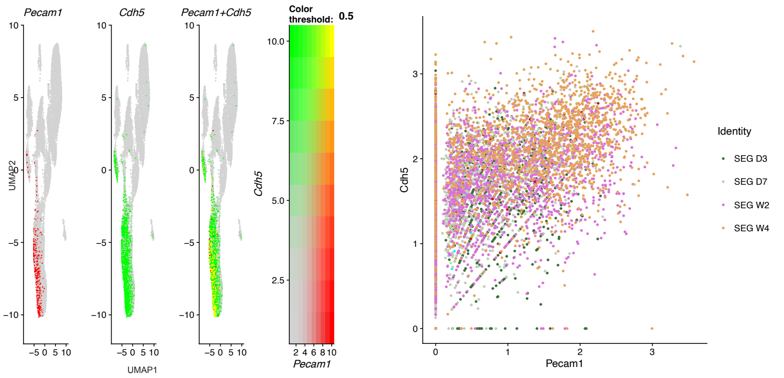

f **g** **h** **i**

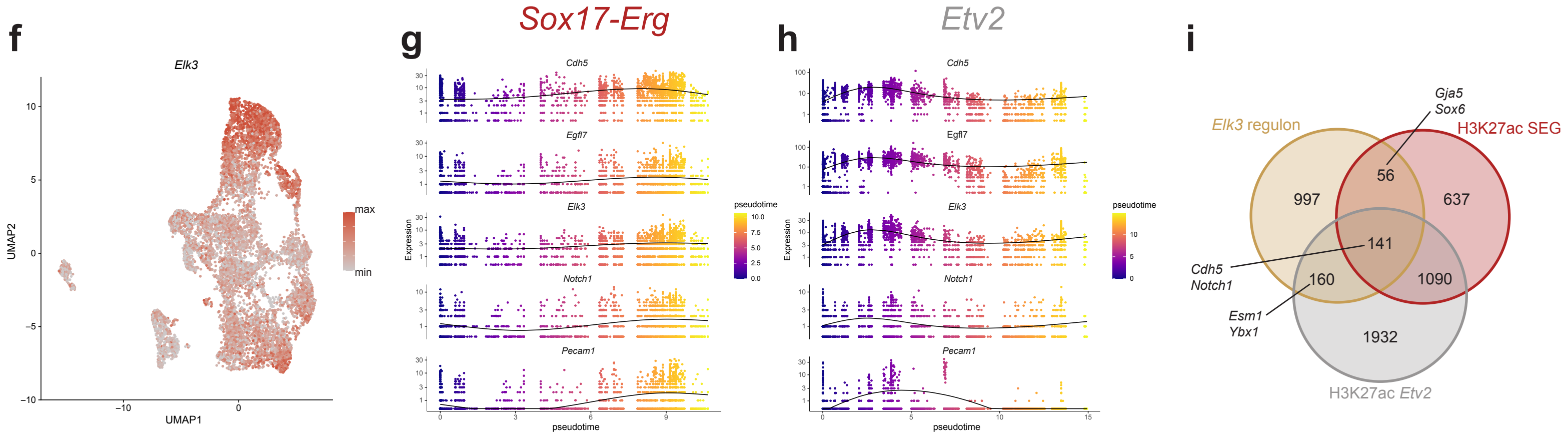

a, Quality control metrics of adult CF reprogramming scRNAseq by sample type and timepoint
b, Percent cluster composition table of samples with highest sample type in cluster bolded.
c, Co-Expression Plot of *Cdh5* and *Pecam1*
d, Scatter plot of *Cdh5* and *Pecam1* of SEG clusters 1,3,4,5 labeled by sample timepoint.
e, In silico gene perturbation of *Elk3* using CellOracle
f, *Elk3* expression in neonatal iECs and cardiac fibroblasts
g, Pseudotime expression of *Elk3*, select components of *Elk3* regulon, and *Pecam1* in *Sox17+Erg*
h, Pseudotime expression of *Elk3*, select components of *Elk3* regulon, and *Pecam1* in *Etv2*
i, Venn diagram comparison of SEG *Elk3* regulon genes, and H3K27ac SEG and *Etv2* upregulated genes.

Supplementary Figure 13: Higher magnification images of in vivo Reprogramming

a

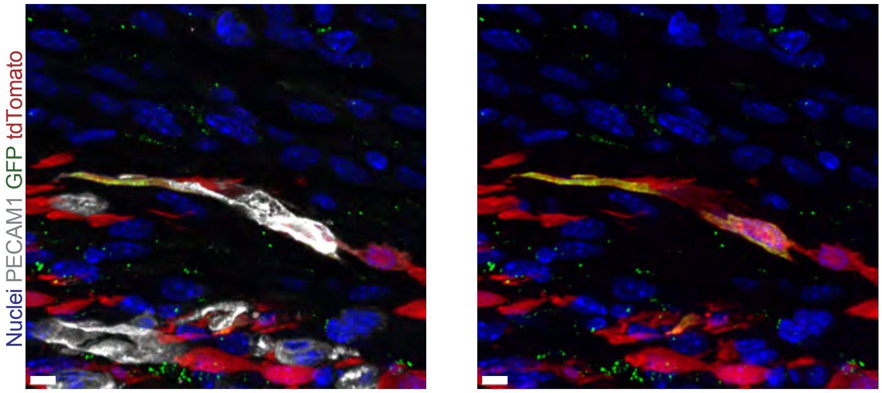

b

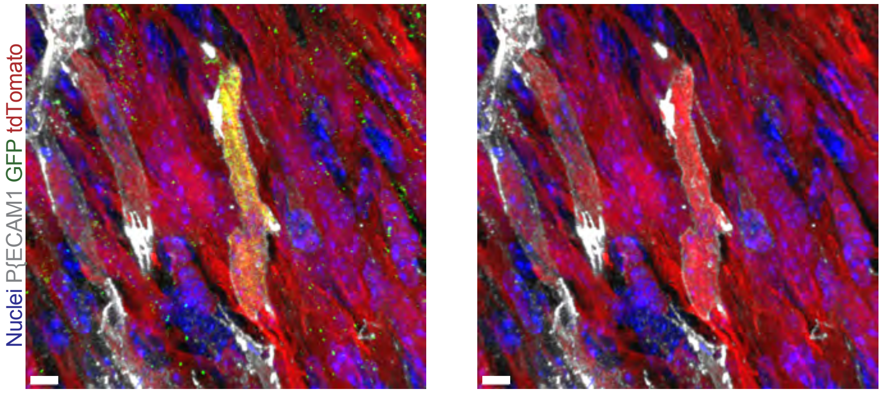

a,**b,** Additional high magnification images of SEG iECs (scale bars 5 microns)

Supplementary Figure 14: Flow Cytometry Gating Strategy Example

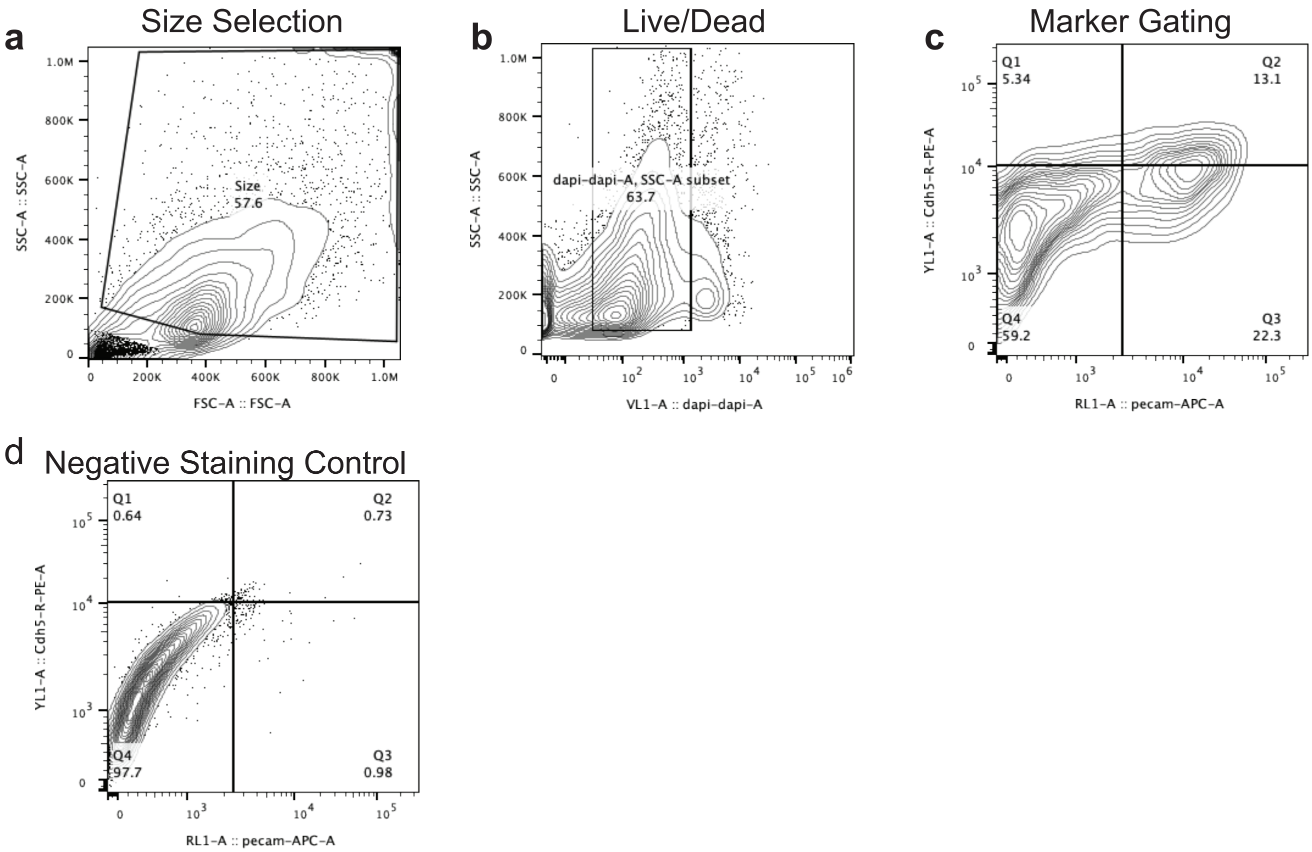

a, Gating of cells using SSC and FSC
b, Gating of Live cells from Dead cells using DAPI
c, Example of gating of cells for CDH5-PE and PECAM1-APC using sample cells
d, Example of negative staining control sample with the CDH5-PE and PECAM1-APC gating
