# Supplementary material for: Direct conversion of cardiac fibroblasts into endothelial-like cells using Sox17 and Erg
Source: Nat Commun. 2024 May 16;15:4170. doi: 10.1038/s41467-024-48354-6 (PMC11098819; doi:10.1038/s41467-024-48354-6)
Supplement: Supplementary file 3 — Description of additional supplementary files [file 41467_2024_48354_MOESM3_ESM.pdf]

Description of Additional Supplementary Files

Supplementary Data 1: Marker genes of neonatal iEC clusters (Seurat FindAllMarkers Wilcoxon Rank Sum test)
