# Supplementary material for: Direct conversion of cardiac fibroblasts into endothelial-like cells using Sox17 and Erg
Source: Nat Commun. 2024 May 16;15:4170. doi: 10.1038/s41467-024-48354-6 (PMC11098819; doi:10.1038/s41467-024-48354-6)
Supplement: Supplementary file 5 — Reporting summary [file 41467_2024_48354_MOESM5_ESM.pdf]

nature portfolio

Corresponding author(s): Li Qian, Ph.D.

Last updated by author(s): 3/21/24

Reporting Summary

Nature Portfolio wishes to improve the reproducibility of the work that we publish. This form provides structure for consistency and transparency in reporting. For further information on Nature Portfolio policies, see our Editorial Policies and the Editorial Policy Checklist.

Please do not complete any field with "not applicable" or n/a. Refer to the help text for what text to use if an item is not relevant to your study.
For final submission: please carefully check your responses for accuracy; you will not be able to make changes later.

Statistics

For all statistical analyses, confirm that the following items are present in the figure legend, table legend, main text, or Methods section.

n/a	Confirmed	
☐	☑	The exact sample size (*n*) for each experimental group/condition, given as a discrete number and unit of measurement
☐	☑	A statement on whether measurements were taken from distinct samples or whether the same sample was measured repeatedly
☐	☑	The statistical test(s) used AND whether they are one- or two-sided<br>*Only common tests should be described solely by name; describe more complex techniques in the Methods section.*
☐	☑	A description of all covariates tested
☐	☑	A description of any assumptions or corrections, such as tests of normality and adjustment for multiple comparisons
☐	☑	A full description of the statistical parameters including central tendency (e.g. means) or other basic estimates (e.g. regression coefficient) AND variation (e.g. standard deviation) or associated estimates of uncertainty (e.g. confidence intervals)
☐	☑	For null hypothesis testing, the test statistic (e.g. *F*, *t*, *r*) with confidence intervals, effect sizes, degrees of freedom and *P* value noted<br>*Give P values as exact values whenever suitable.*
☑	☐	For Bayesian analysis, information on the choice of priors and Markov chain Monte Carlo settings
☑	☐	For hierarchical and complex designs, identification of the appropriate level for tests and full reporting of outcomes
☑	☐	Estimates of effect sizes (e.g. Cohen's *d*, Pearson's *r*), indicating how they were calculated

Our web collection on statistics for biologists contains articles on many of the points above.

Software and code

Policy information about availability of computer code

Data collection	EVOS M7000 Imaging Software, Zeiss Zen Blue for LSM900, Attune NxT Flow Cytometer Software
Data analysis	FlowJo, CellProfiler, Imaris, AutoQuant, FIJI, QuPath, GraphPad Prism, Seurat (5.0.0), ClusterProfiler (4.10.1), CellID, Monocle3, SeuratWrappers, tricycle (1.10.0), CellChat, cellranger, CellOracle (0.17.1), DESeq2 (1.42.1), HOMER, ChIPseeker(1.38.0), Deeptools (3.5.5), SEACR(1.3). Code can be made available upon request.

For manuscripts utilizing custom algorithms or software that are central to the research but not yet described in published literature, software must be made available to editors and reviewers. We strongly encourage code deposition in a community repository (e.g. GitHub). See the Nature Portfolio guidelines for submitting code & software for further information.

Data

Policy information about availability of data

All manuscripts must include a data availability statement. This statement should provide the following information, where applicable:

- Accession codes, unique identifiers, or web links for publicly available datasets
- A description of any restrictions on data availability
- For clinical datasets or third party data, please ensure that the statement adheres to our policy

All genomics data is accessible under NIH GEO Database GSE218418

Research involving human participants, their data, or biological material

Policy information about studies with human participants or human data. See also policy information about sex, gender (identity/presentation), and sexual orientation and race, ethnicity and racism.

Reporting on sex and gender	N/A
Reporting on race, ethnicity, or other socially relevant groupings	N/A
Population characteristics	N/A
Recruitment	N/A
Ethics oversight	N/A

Note that full information on the approval of the study protocol must also be provided in the manuscript.

Field-specific reporting

Please select the one below that is the best fit for your research. If you are not sure, read the appropriate sections before making your selection.

☑ Life sciences ☐ Behavioural & social sciences ☐ Ecological, evolutionary & environmental sciences

For a reference copy of the document with all sections, see nature.com/documents/nr-reporting-summary-flat.pdf

Life sciences study design

All studies must disclose on these points even when the disclosure is negative.

Sample size	An sample size (n) of three was determined to be the minimum sample size for any validation studies. Experimental sample size was determined by researching the industry standard for each applicable experiment.
Data exclusions	Data was only excluded if a replicate was an outlier two or more other replicates. If data was excluded, additional replicates would be performed with necessary controls in order to reach necessary sample size.
Replication	All experiments first underwent pilot experiments to ensure optimal conditions and working controls. Replicates of control and experiment conditions were done in parallel with at least three replicates per condition for non-sequencing experiments. All experiments contained control samples to ensure working reagents. All collected replicates are reported..
Randomization	When applicable, sample regions for quantification were selected using microscope automation.
Blinding	All quantification was performed in a blinded manner by computational software (i.e. CellProfiler, FIJI) and by researcher.

Behavioural & social sciences study design

All studies must disclose on these points even when the disclosure is negative.

Study description	
Research sample	
Sampling strategy	
Data collection	
Timing	
Data exclusions	
Non-participation	
Randomization	

Ecological, evolutionary & environmental sciences study design

All studies must disclose on these points even when the disclosure is negative.

Study description	
Research sample	
Sampling strategy	
Data collection	
Timing and spatial scale	
Data exclusions	
Reproducibility	
Randomization	
Blinding	

Did the study involve field work? ☐ Yes ☐ No

Field work, collection and transport

Field conditions	
Location	
Access & import/export	
Disturbance	

Reporting for specific materials, systems and methods

We require information from authors about some types of materials, experimental systems and methods used in many studies. Here, indicate whether each material, system or method listed is relevant to your study. If you are not sure if a list item applies to your research, read the appropriate section before selecting a response.

Materials & experimental systems

n/a	Involved in the study	
☐	☑	Antibodies
☐	☑	Eukaryotic cell lines
☑	☐	Palaeontology and archaeology
☐	☑	Animals and other organisms
☑	☐	Clinical data
☑	☐	Dual use research of concern
☑	☐	Plants

Methods

n/a	Involved in the study	
☐	☑	ChIP-seq
☐	☑	Flow cytometry
☑	☐	MRI-based neuroimaging

Antibodies

Antibodies used	anti-CD31 (R&D #AF3628); anti-Cdh5 (R&D #AF1002); anti-GFP (Aves #GFP-1020). Donkey anti-Goat 568 (ThermoFisher #A-11057); Donkey anti-Chicken 647(Jackson Immunoresearch (#703-605-155); Donkey anti-Chicken 488 (Jackson Immunoresearch #703-545-155), H3K27ac (Abcam #4729). Cdh5-PE (ThermoFisher #12-1441-82), Pecam-APC (R&D FAB3628A).
Validation	All primary antibodies were select due to use by previously published peer reviewed articles. Antibodies were then validated by comparing primary plus secondary staining to negative control (secondary only).

Eukaryotic cell lines

Policy information about cell lines and Sex and Gender in Research

Cell line source(s)	platE cells- Cell BioLabs.
Authentication	platE cells were validated by retroviral GFP production.
Mycoplasma contamination	none
Commonly misidentified lines (See ICLAC register)	none

Palaeontology and Archaeology

Specimen provenance	
Specimen deposition	
Dating methods	

☐ Tick this box to confirm that the raw and calibrated dates are available in the paper or in Supplementary Information.

Ethics oversight	

Note that full information on the approval of the study protocol must also be provided in the manuscript.

Animals and other research organisms

Policy information about studies involving animals; ARRIVE guidelines recommended for reporting animal research, and Sex and Gender in Research

Laboratory animals	C57BL/6J mice (Jackson Strain#000664) (ages: Day0-Day4, 6-12 weeks old), ROSA26-tdTomato (#007914) (age: 6-12weeks). Tcf21-CreERT2 (gift from Song et. al 2012)(age: 6-12weeks). SCID (Charles River #236)(age: 6-12weeks).
Wild animals	none
Reporting on sex	Gender of male and female mice for in vivo studies was recorded.
Field-collected samples	none
Ethics oversight	All animal studies were approved by UNC IACUC (#21-187) and IBC.

Note that full information on the approval of the study protocol must also be provided in the manuscript.

Clinical data

Policy information about clinical studies
All manuscripts should comply with the ICMJE guidelines for publication of clinical research and a completed CONSORT checklist must be included with all submissions.

Clinical trial registration	
Study protocol	
Data collection	
Outcomes	

Dual use research of concern

Policy information about dual use research of concern

Hazards

Could the accidental, deliberate or reckless misuse of agents or technologies generated in the work, or the application of information presented in the manuscript, pose a threat to:

No	Yes	
☐	☐	Public health
☐	☐	National security
☐	☐	Crops and/or livestock
☐	☐	Ecosystems
☐	☐	Any other significant area

Experiments of concern

Does the work involve any of these experiments of concern:

No	Yes	
☐	☐	Demonstrate how to render a vaccine ineffective
☐	☐	Confer resistance to therapeutically useful antibiotics or antiviral agents
☐	☐	Enhance the virulence of a pathogen or render a nonpathogen virulent
☐	☐	Increase transmissibility of a pathogen
☐	☐	Alter the host range of a pathogen
☐	☐	Enable evasion of diagnostic/detection modalities
☐	☐	Enable the weaponization of a biological agent or toxin
☐	☐	Any other potentially harmful combination of experiments and agents

Plants

Seed stocks	
Novel plant genotypes	
Authentication	

ChIP-seq

Data deposition

☑ Confirm that both raw and final processed data have been deposited in a public database such as GEO.

☑ Confirm that you have deposited or provided access to graph files (e.g. BED files) for the called peaks.

Data access links<br>*May remain private before publication.*	GSE218415
Files in database submission	control rep1, control rep2, Etv2 rep1, Etv2 rep2, SEG rep1, SEG rep2, neonatal EC rep1, neonatal EC rep2.
Genome browser session<br>(e.g. UCSC)	

Methodology

Replicates	2 replicates per sample type
Sequencing depth	400M (Novaseq 2000) was used for pooled samples.
Antibodies	H3K27ac (Abcam #4729)
Peak calling parameters	SEACR top 0.01%
Data quality	Assessed using standard CUT&Tag data analysis pipeline
Software	R studio with standard CUT&Tag data analysis pipeline

Flow Cytometry

Plots

Confirm that:

☑ The axis labels state the marker and fluorochrome used (e.g. CD4-FITC).

☑ The axis scales are clearly visible. Include numbers along axes only for bottom left plot of group (a 'group' is an analysis of identical markers).

☑ All plots are contour plots with outliers or pseudocolor plots.

☑ A numerical value for number of cells or percentage (with statistics) is provided.

Methodology

Sample preparation	Cells are detached from cell culture plate using Accutase (#A6964-100mL) and a equal volume of EGM media is added to the detached cells. The cells are then centrifuged at 300xg for 5 minues and the pelleted cells are resuspended in 100uL of FACS Buffer (0.5% BSA in PBS) with 10uL of APC-conjugated Pecam1 antibody (R&D FAB3628A) or 5uL of PE-conjugated Cdh5 antibody (ThermoFisher #12-1441-82). Cell are incubated in antibody solution for 30 minutes at 4□C. The samples are then washed with 1mL of FACS buffer and centrifuged at 300xg for 5 minutes (repeated 3 times). After the last wash, the cells are filtered through a 40micron strainer, briefly treated with DAPI to label dead cells, and then analyzed using ThermoFisher Atunne NxT.
Instrument	Attune NxT Flow Cytometer
Software	FlowJo, Attune NxT Flow Cytometer Software
Cell population abundance	Thresholds were set so that a over 10,000 cells would be collected for each sample during initial collection.
Gating strategy	Size selection, Live/Dead, then Dual Marker Expression using negative control.

☑ Tick this box to confirm that a figure exemplifying the gating strategy is provided in the Supplementary Information.

Magnetic resonance imaging

Experimental design

Design type	
Design specifications	
Behavioral performance measures	

Imaging type(s)	
Field strength	
Sequence & imaging parameters	
Area of acquisition	

Diffusion MRI ☐ Used ☐ Not used

Preprocessing

Preprocessing software	
Normalization	
Normalization template	
Noise and artifact removal	
Volume censoring	

Statistical modeling & inference

Model type and settings	
Effect(s) tested	

Specify type of analysis: ☐ Whole brain ☐ ROI-based ☐ Both

Statistic type for inference

(See Eklund et al. 2016)

Correction

Models & analysis

n/a	Involved in the study
☐	☐ Functional and/or effective connectivity
☐	☐ Graph analysis
☐	☐ Multivariate modeling or predictive analysis

Functional and/or effective connectivity

Graph analysis

Multivariate modeling and predictive analysis

This checklist template is licensed under a Creative Commons Attribution 4.0 International License, which permits use, sharing, adaptation, distribution and reproduction in any medium or format, as long as you give appropriate credit to the original author(s) and the source, provide a link to the Creative Commons license, and indicate if changes were made. The images or other third party material in this article are included in the article's Creative Commons license, unless indicated otherwise in a credit line to the material. If material is not included in the article's Creative Commons license and your intended use is not permitted by statutory regulation or exceeds the permitted use, you will need to obtain permission directly from the copyright holder. To view a copy of this license, visit http://creativecommons.org/licenses/by/4.0/
